# Insight on a comprehensive profile of volatile compounds of *Chlorella vulgaris* extracted by two “green” methods

**DOI:** 10.1002/fsn3.831

**Published:** 2019-02-11

**Authors:** Céline Lafarge, Nathalie Cayot

**Affiliations:** ^1^ AgroSup Dijon UMR PAM A02.102 Université Bourgogne Franche‐Comté Dijon France

**Keywords:** *Chlorella vulgaris*, extraction, green solvent, SPME, ultrasound, volatile compound

## Abstract

Some green extraction methods were selected and tested for the extraction of volatile compounds from different samples of the microalga *Chlorella vulgaris:* ultrasound‐assisted liquid–liquid extraction using environment‐friendly solvents (LLE) and solid‐phase microextraction (SPME). The obtained profiles of volatile chemical compounds were different. Only one molecule was found in common to both extractions. Using the SPME method, the main chemical classes of identified volatile compounds were sulfuric compounds, aldehydes, and alcohols. Using the LLE method, the volatile profile was more balanced with alkanes, fatty acids, terpenes, alcohols, and aldehydes. Multivariate data analyses permitted discrimination among samples. Additionally, the relationship between the physicochemical properties of identified volatile compounds and the methods of extraction was studied. The results showed that the LLE extraction allowed the extraction of volatile compounds having a high boiling point (>160°C) and a high log P (>3). The SPME method was more effective to extract volatile compounds with a low boiling point (<160°C) and a low log P (<3). It is thus necessary to combine several extraction methods to obtain a complete view of the volatile profile for microalgae samples.

## 1 INTRODUCTION

Nowadays, in Europe, food, pharmaceutical, and cosmetic industries pay a special attention to microalgae (Spolaore, Joannis‐Cassan, Duran, & Isambert, 2006; Vigani et al., 2015) that are promising source of nutrients. Among them, *Chlorella vulgaris* is commonly used. This microalga is interesting because of nutritional and health benefits: remarkable richness in proteins, lipids, polysaccharides, pigments, vitamins, and antioxidants. Furthermore, *Chlorella vulgaris* shows potential activity as preservative (Borowitzka, 2013; Draaisma et al., 2013; Safi, Zebib, Merah, Pontalier, & Vaca‐Garcia, 2014; Vaz, Moreira, Morais, & Costa, 2016).

Nevertheless, the use of microalgae as food ingredient is still poorly developed in Europe due to a low demand and a strict European regulation (Batista et al., 2017). To show the potential of microalgae as food ingredient, some studies have been published on healthy food products containing microalgae such as vegetarian food gels (Batista et al., 2012), pasta (De Marco Rodríguez, Steffolani, Martínez, & León, 2014; Fradique et al., 2010), cookies (Batista et al., 2017), and bread (Kadam & Prabhasankar, 2010). To improve the knowledge on microalgae, studies have been performed to determine the impact of their incorporation in food products and their digestibility (Batista et al., 2012; Martínez‐Sanz, Gómez‐Mascaraque, & López‐Rubio, 2017), (De Marco Rodríguez et al., 2014).

The incorporation of microalgae into foodstuffs can also modify the overall flavor leading to desirable or, on the contrary, to unwanted organoleptic properties. Some data are available on the profile in volatile compounds of microalgae (Abdel‐Baky, Shallan, El‐ Baroty, & El‐Baz, 2002; Isleten Hosoglu, 2018; Van Durme, Goiris, De Winne, De Cooman, & Muylaert, 2013), and on the impact of the growth phases (Zhou et al., 2017), the process, and the conditions of storage on this profile (Santos, Fernandes, Wagner, Jacob‐Lopes, & Zepka, 2016). Depending on culture and environmental conditions, the microalgae are able to produce a variety of volatile compounds (Isleten Hosoglu, 2018; Van Durme et al., 2013).

Nevertheless, few are the reported studies concerning the analysis of the whole profile of volatile compounds produced by microalgae (Abdel‐Baky et al., 2002; Santos et al., 2016; Van Durme et al., 2013; Zhou et al., 2017). Rzama, Benharref, Arreguy, and Dufourc (1995) performed the extraction of an essential oil from *Chlorella vulgaris*. After tentative identification by gas chromatography–mass spectrometry (GC‐MS), they concluded that most of the volatile compounds identified had low odor thresholds and that esters and alcohols, representing about 40% of the volatile oil, were responsible for unpleasant odors. More recently, Isleten Hosoglu (2018) used SPME followed by GC‐MS and olfactometry to study the aroma profile of different microalgae among which *Chlorella vulgaris*. The results of PCA showed that *Chlorella vulgaris* was ill represented in this study.

So, there is still a need to acquire knowledge on the volatile compounds profile of microalgae.

Different methods, classified into two main groups, can be used to extract volatile compounds: classical extraction methodologies such as liquid–liquid extraction (LLE) and headspace techniques.

With LLE, all volatile compounds can be extracted in one extraction step if the organic solvent is appropriate. Nevertheless, in relation to the nature of the sample and the nature of target molecule to extract, several extraction steps with different organic solvents can be essential. Moreover, a concentration step is sometimes necessary before chromatographic analysis with potential loss of volatile compounds and generation of artifact. Improvements can be made. For example, LLE can be improved using ultrasounds that accelerate heat and mass transfer and consequently enhance the extraction and yield recovery of target molecules (Chemat et al., 2017). Additionally, ultrasound‐assisted extraction can be scaled up to industrial production (Michalak & Chojnacka, 2014). To go further, safety and environmental aspects must be taken into account. The challenge of green extraction is to “*design extraction processes which will reduce energy consumption, allows use of alternative solvents and renewable natural products, and ensure a safe and high quality extract/product* (Chemat et al., 2017).”. Several works have been conducted to find “green extraction solvents”( Armenta & de la Guardia, 2016; Cayot, Lafarge, Bou‐Maroun, & Cayot, 2016; Li, Fabiano‐Tixier, Ginies, & Chemat, 2014; Li, Fine et al., 2014) or “green method of extraction” (Filly et al., 2014; Sanchez‐Prado, Garcia‐Jares, Dagnac, & Llompart, 2015). LLE using the azeotrope “propan‐2‐one/cyclopentane” was reported as a suitable one for the extraction of volatile compounds (Cayot et al., 2016).

Considering headspace techniques, SPME (solid‐phase microextraction) is one of the most widely used. The SPME method combines sampling and sample preparation in one step (Wardencki, Michulec, & Curyło, 2004). This method is based on the adsorption of molecules onto a silica fiber which is coated with a polymer specifically selected for the target molecules (Arthur & Pawliszyn, 1990). The disadvantage of the SPME method is (a) that it is less sensitive for the extraction of molecules with low volatility (Sánchez‐Palomo, Alañón, Díaz‐Maroto, González‐Viñas, & Pérez‐Coello, 2009) and (b) the SPME method is also based on a double equilibrium: liquid–gas partition and gas‐fiber equilibrium. However, for extraction of molecules with low volatility, the direct immersion SPME (DI‐SPME) is the second most common mode used. In this case, the fiber is directly placed into the solution. Whatever the technique (DI‐SPME or HS‐SPME), the performance of SPME method is influenced by the nature of the selected fiber as the fiber coating has the most important direct impact on the extraction efficiency of a typical SPME approach. The different fiber materials offer a range of polarity of the coating to extract volatile and semi‐volatile compounds. The materials have also been combined to create fibers able to sample compounds with a wider range of properties than if a single material had been used.

Nevertheless, compared to the LLE, the SPME method has the advantage to present a high sensitivity and to reduce extraction time. It is a solvent‐free extraction, without any sample preparation.

For the present study, *Chlorella vulgaris* was chosen as a model of microalgae. The aim of this work was to obtain a complete profile of volatile compounds from different samples of *Chlorella vulgaris* using two complementary extraction techniques: the SPME method and an ultrasound‐assisted liquid–liquid extraction. These methods were chosen because they are rapid, simple, and environmental‐friendly techniques.

## 2 MATERIALS AND METHODS

### 2.1 Materials

Six different samples of *Chlorella vulgaris* were used (kindly given by Roquette, France). The microalgae were cultivated under heterotrophic conditions in controlled bioreactors. The medium of cultivation, the growth, and the processing conditions were varied to produce six different samples. After harvesting, the six different samples of *Chlorella vulgaris* coming from six different batches were spray‐dried. It is known that the biochemical composition of microalgae depends on (a) the macro‐ and micronutrients that are used to prepare culture media and (b) on their culture conditions (Vaz et al., 2016). For reference purposes, Table 1 regroups the variations of the main components of *Chlorella vulgaris* reported in the literature.

**Table 1:** Variations of main components of C*hlorella vulgaris* reported in the literature

	% dry weight	References
Total proteins	42–58	Safi et al. (2014)
	25–30	Van Durme et al. (2013)
Lipids	5–40	Safi et al. (2014)
	12	Van Durme et al. (2013)
Carbohydrates	12–55	Safi et al. (2014)
	6	Batista et al. (2017)
	8	Gamero et al. (2013)
Ash	9	Batista et al. (2017)
	6.3	Gamero et al. (2013)

The samples were labeled A, B, C, D, E, and F. The samples were stored at ambient temperature in dark glass bottles. Immediately before analysis, the samples were diluted in deionized water to obtain 85 g dry matter / L.

For the headspace solid‐phase microextraction, naphthalene‐d8 (Supelco, CAS number 1146‐65‐2) was diluted in 2,2,4‐trimethylpentane (Sigma‐Aldrich, purity 99%, CAS number 540‐84‐1) at 12.10^−3^ g/L and added as internal standard.

For ultrasound‐assisted liquid–liquid extraction, the following reagents were used: anhydrous sodium sulfate (VWR, CAS 7757‐82‐6) to dehydrate liquid extracts, azeotrope “propan‐2‐one (Sigma, purity >99.5%, CAS number 067‐64‐1) and cyclopentane (Sigma‐Aldrich, purity 98%, CAS number 287‐92‐3)” as the extraction solvent, naphthalene‐d8 (Supelco, CAS number 1146‐65‐2) diluted at 2 g/L in azeotrope “propan‐2‐one/cyclopentane” as the internal standard.

### 2.2 Extraction methods of volatile compounds

#### 2.2.1 Headspace solid‐phase microextraction (HS‐SPME)

This analysis was carried out using a three‐phase fiber (divinylbenzene (DVB)/carboxen (CAR)/polydimethylsiloxane (PDMS), 50/30 μm, Supelco). In order to optimize the extraction, a preliminary study was done to choose the best extraction parameters (choice of fiber, extraction parameters). In fact, in SPME, the amount of molecule extracted onto the fiber depends not only on the polarity and thickness of the stationary phase, but also on the extraction time and the concentration of interest molecules in the sample. In general, volatile extraction is best achieved when the polarity of the fiber matches the polarity of the interest molecules (i.e., non‐polar fibers for non‐polar molecules and polar fibers for polar molecules). Consequently, different types of commercial SPME fibers with different coating have been tested in a preliminary study. Better results were obtained using 50/30 μm DVB/CAR/PDMS fiber. This triple‐phase DVB‐CAR‐PDMS SPME fiber is extensively used (Heaven & Nash, 2012) and is recommended by suppliers (Supelco Sigma, 2018). Before use, the fiber was conditioned in accordance with the manufacturer's recommendations.

The selected extraction conditions were the followings: 2 ml of sample in a 20 ml vial was incubated in a water bath at 40°C during 30 min under magnetic stirring. 200 μl of internal standard was added to the sample before the incubation of the sample. Then, the fiber was exposed to the sample headspace during 30 min and was desorbed for 15 min into GC‐MS. The analyses were done in triplicate.

#### 2.2.2 Ultrasound‐assisted liquid–liquid extraction (ultrasound‐assisted LLE)

The ultrasound‐assisted LLE was done as described by Cayot et al. (2016) 5 g of sample, 25 ml of the extraction solvent (azeotrope “propan‐2‐one / cyclopentane” in a molar ratio of 59.55 / 40.45) and 100 μl of internal standard were strongly stirred for 5 min. Then, the flasks were placed in an ultrasound bath (Bransonic Mod 5210, Branson Europe B.V.) for 10 min at an ultrasound fixed‐frequency of 47 kHz ± 6%. Immediately after sonication, the suspensions were removed from the ultrasound bath and centrifuged (6660 *g*, 10 min, 20°C). The supernatant (organic phase) was taken and dehydrated using sodium sulfate. This extract was then filtered using glass wool and concentrated using Rotavapor^®^ (Buchi apparatus, 40°C, 120 mbar) up to 50 % of the extract volume. These concentrated extracts were analyzed by GC‐MS. Extractions were done in triplicate.

### 2.3 Gas chromatography–mass spectrometry analysis

The SPME fibers were analyzed with a mass spectrometry (Shimadzu QP2010 + , electronic impact at 70 eV) paired with a Shimadzu 2010 gas chromatograph fitted with a split/splitless injector (240°C). The chromatograph was equipped with a capillary column PEG of 30 m × 0.32 mm (J&W Scientific). Film thickness was 0.50 μm. Helium was used as vector gas at a rate of 1.5 ml/min (average velocity of 44 cm/s). The temperature of the oven was increased from 40 to 130°C at 3°C/min, then from 130 to 250°C at 10°C/min, and finally held at 250°C for 5 min. The injection mode was splitless.

The liquid extracts were analyzed with a mass spectrometry (HP5973, electronic impact at 70 eV, temperature source at 230°C) paired with a Hewlett–Packard 6980 gas chromatograph fitted with a split/splitless injector (240°C). The chromatograph was equipped with a capillary column DB WAX of 30 m × 0.32 mm (J&W Scientific). Film thickness was 0.25 μm. Helium was used as vector gas at a rate of 1.5 ml/min (44 cm/s). One μl of each extract was injected automatically. The temperature of the oven was increased from 35 to 120°C at 5°C/min, then from 120 to 240°C at 20°C/min, and finally held at 240°C for 10 min. The injection mode was split mode with a split ratio of 5.

Whatever the analytical method used, spectrometry selected ion monitoring method (SIM method) was used for molecules detection. The mass spectrometer scanned from *m*/*z* 29 to 500. The twenty highest peaks were kept and corresponding volatile compounds were tentatively identified by matching their spectral fragmentation with those provided by the mass spectral library of the National Institute of Standards and Technology (NIST) and the Wiley Registry (WILEY).

In addition, for each volatile compounds obtained from liquid extracts, linear retention index (LRI) was calculated using the retention times of a standard mixture of C7‐C30 saturated alkanes (Sigma‐Aldrich) and compared with the LRI values published in the literature for columns with the same polarity.

Relative semi‐quantification was done using the internal standard.

### 2.4 Statistical analysis

For each extraction method, principal component analysis (PCA) (Statistica V8 software) was used to illustrate the differences among the six microalgae samples.

## 3 RESULTS AND DISCUSSION

### 3.1 SPME

Table 2 shows the identification and semi‐quantification of volatile compounds from the six samples of *Chlorella vulgaris* obtained by the SPME method. A total of nine volatile compounds were identified including three aldehydes, one sulfuric compound, four terpenes, and one alcohol.

**Table 2:** Volatile compounds extracted by SPME from six spray‐dried samples of C*hlorella vulgaris*: average value (μg / L) with standard deviation and odors descriptors (According to(The_good_scents_company, 2016))

Compound (synonym)	CAS Number	Odor descriptors	Spray‐dried samples of C*hlorella vulgaris*					
			A	B	C	D	E	F
Aldehydes								
3‐Methylbutanal (Isovaleraldehyde)	590‐86‐3	Ethereal aldehydic chocolate peach fatty	4 ± 1	5 ± 1	45 ± 2	736 ± 13	1.8 ± 0.2	1.4 ± 0.5
Hexanal	66‐25‐1	Fresh green fatty aldehydic grass leafy fruity sweaty	251 ± 25	328 ± 80	118 ± 5	104 ± 11	97 ± 77	230 ± 85
Benzaldehyde	100‐52‐7	Strong sharp sweet bitter almond cherry	10 ± 0.9	27 ± 1	25 ± 3	11 ± 1	11 ± 1	9 ± 3
Total			265	360	188	851	110	240
Sulfuric compound								
Dimethyl disulfide	624‐92‐0	Sulfurous vegetable cabbage onion	29 ± 5	53 ± 23	4 ± 2	8 ± 12	111 ± 7	99 ± 22
Terpenes								
β‐Cyclocitral	432‐25‐7	Tropical saffron herbal clean rose oxide sweet tobacco damascone fruity	14 ± 2	18 ± 1	14 ± 1.5	5 ± 0.5	15 ± 2	17 ± 1
4‐(2,6,6‐Trimethyl‐2‐cyclohexenyl)‐3‐buten‐2‐one(α‐Ionone)	127‐41‐3	Sweet woody floral violet orris tropical fruity	8 ± 1	9 ± 1	8 ± 0.2	2 ± 0.2	9 ± 1	9 ± 0.3
4‐(2,6,6‐Trimethyl‐1‐cyclohexenyl)‐3‐buten‐2‐one (β‐Ionone)	79‐77‐6 14901‐07‐6	Dry powdery floral woody orris sweet fruity berry tropical beeswax	21 ± 5	23 ± 3	14 ± 0.4	6 ± 0.4	17 ± 1	21 ± 0.6
p‐Cresol	106‐44‐5	Phenolic narcissus animal mimosa	0.04 ± 0.01	0.6 ± 0.1	0.14 ± 0.00	0.07 ± 0.00	0.10 ± 0.01	0.18 ± 0.02
Total			43	51	36	13	41	47
Alcohols								
1‐Octen‐3‐ol	3391‐86‐4	Mushroom earthy green oily fungal raw chicken	5 ± 0.4	8 ± 0.6	7 ± 0.9	5 ± 0.6	6 ± 0.5	22 ± 4

The most abundant chemical class of volatile compounds detected in the microalgae samples was aldehyde. The total aldehyde content ranged from 110 μg/L (sample E) to 851 μg/L (sample D). For each sample, hexanal was identified as the most prevalent volatile compound with high amount considering the whole profile of samples. With a mean value of 188 μg/L, hexanal can be considered as a biomarker of *Chlorella vulgaris*, even if it is not a very specific volatile compound. Moreover, with a recognition threshold in water of 4.5 μg / L (Belitz, Burghagen, Grosch, & Schieberle, 2004; Bugaud & Alter, 2016), this molecule characterized by an odor of fresh green grass(The_good_scents_company, 2017) could contribute to the aroma of *Chlorella vulgaris*. This result is in accordance with the results of Van Durme et al. (2013). The linear aldehydes such as hexanal are often derived from chemical lipid oxidation (Santos et al., 2016; Van Durme et al., 2013) and/or autoxidation or enzymatic oxidation of linoleic acid (Belitz et al., 2004; Isleten Hosoglu, 2018; Jayasena, Ahn, Nam, & Jo, 2013; Zhou et al., 2017). Aromatic aldehydes (3‐methylbutanal and benzaldehyde) are typically formed due to enzymatic oxidation of lipid and protein oxidation (Van Durme et al., 2013). Other authors reported that the probable origin of 3‐methylbutanal could be Strecker degradation of leucine (Belitz et al., 2004; Rainer Cremer & Eichner, 2000). For others, benzaldehyde could be formed by the oxidation of benzyl alcohol or by the action of microorganisms on the aromatic amino acids (Van Durme et al., 2013) or alternatively from phenyl acetic acid and p‐hydroxybenzoic acid (Chen, Xu, & Qian, 2013).

Table 2 also shows that dimethyl sulfoxide (sulfur compound) was detected with amounts as different as 4 μg/L for sample C to 111 μg/L for sample E. This volatile compound is characterized by a low recognition threshold in water (0.16–12 μg/L) (Leffingwell, 2017). As this molecule was measured in concentration ranges exceeding its recognition threshold, its characteristic odor of “sulfurous vegetable, cabbage, and onion (The_good_scents_company, 2017)“can contribute to the microalgae flavor too. Anaerobic decomposition of sulfur compounds could be a probable origin of this compound (de Blas et al., 2017). On the other hand, some volatile sulfuric compounds such as methanethiol, dimethyl sulfide potentially initially present can undergo thermal oxidation to form dimethyl disulfide (Isleten Hosoglu, 2018).

The third significant group detected in the microalgae samples was terpenes with a total terpene content varying from 13 μg/L (sample D) to 51 μg/L (sample B). These compounds are products of the carotenoid degradation (Baldermann, Kato, Fleischmann, & Watanabe, 2012; Shumbe et al., 2017).

**Figure 1:**
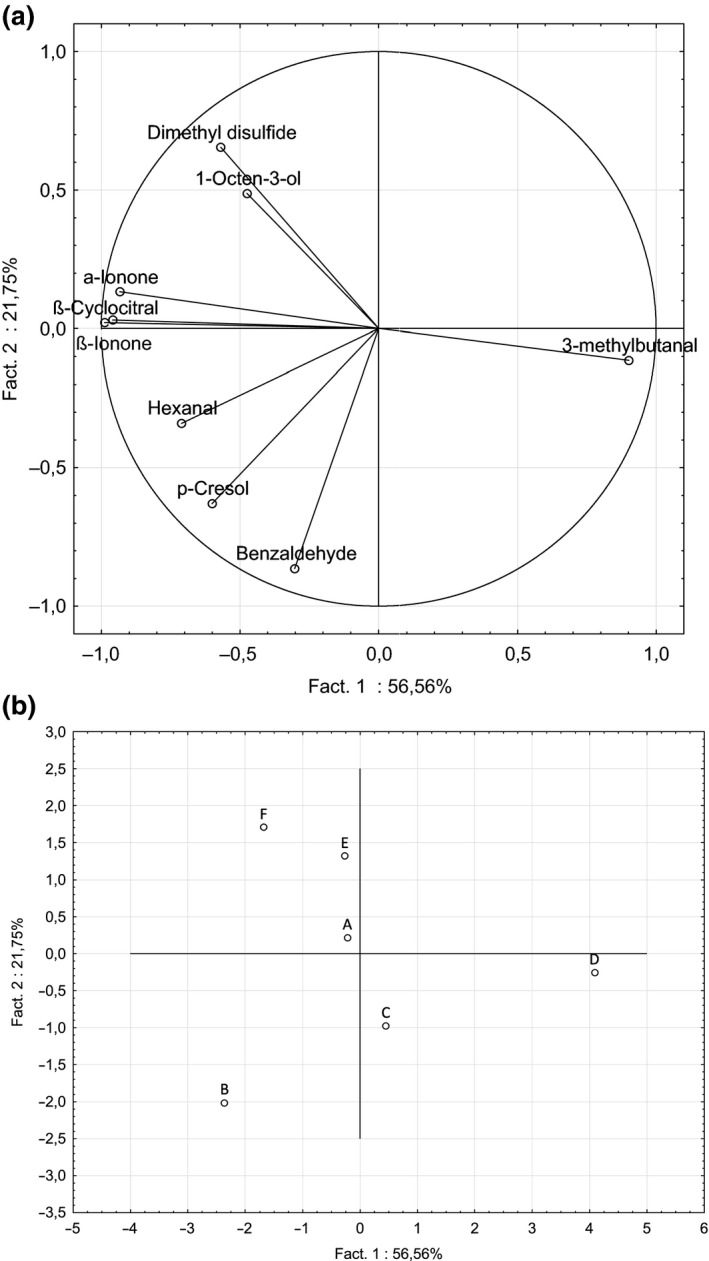
Principal component analysis (PCA) of volatile compounds identified in different samples of C*hlorella vulgaris* obtained by SPME method. (a) correlation circle of variables. (b) two‐dimensional projection of samples

Principal component analysis (PCA) was carried out to display the similarities and differences in volatile compounds detected among samples. The first two principal components were sufficient to explain 79 % of total variance (Figure 1a). The first and second principal components explained a variance of 57 % and 22 %, respectively. The vectors of variables were well represented, close to the correlation circle (Figure 1a). Based on PCA results, the samples could be described by different aroma characteristic. Sample D was well differentiated from the other one (Figure 1b). Sample D was mainly characterized by 3‐methylbutanal (characteristic odor: ethereal aldehydic, fatty (The_good_scents_company, 2017)). Sample B was characterized by benzaldehyde (characteristic odor: bitter almond (The_good_scents_company, 2017)), p‐cresol (characteristic odor phenolic narcissus animal mimosa (The_good_scents_company, 2017)), and hexanal (characteristic odor: fresh green fatty (The_good_scents_company, 2017)). Dimethyl disulfide and 1‐octen‐3‐ol (characteristic odor: mushroom earthy (The_good_scents_company, 2017)) were typical for sample F. In this two‐dimensional representation, it is difficult to describe the volatile compounds profiles of the samples A, E, and C.

With SPME method, the six samples of *Chlorella vulgaris* can be classified into four clusters of volatile compounds profiles. The first cluster composed of sample D, the second one of sample B, the third cluster with sample F, and the fourth with samples A, C, and E. This result could be a result of growth conditions applied leading to differences in the volatile compounds profiles obtained from six samples of *Chlorella vulgaris*.

### 3.2 Ultrasound‐assisted liquid–liquid extraction

A total of 12 volatile compounds were identified using the extracts obtained by ultrasound‐assisted LLE including three aldehydes, three alkanes, three terpenes, two alcohols, and one acid (Table 3).

**Table 3:** Volatile compounds extracted by ultrasound‐assisted liquid–liquid extraction from six spray‐dried samples of C*hlorella vulgaris*: average (μg / L) with standard deviation, experimental retention index (LRI exp.), retention index from literature (LRI lit (Van Durme et al., 2013; Supelco Bulletin 923, 1998).), and odor descriptors (According to(The_good_scents_company, 2016))

Compound (synonym)	CAS Number	LRI exp.	LRI lit.	Odor descriptors	Spray‐dried samples of Chlorella vulgaris					
					A	B	C	D	E	F
Aldehydes										
2‐(3‐Thienyl) butanal	65857‐59‐2	1240	nd	nd	36 ± 4	40 ± 4	44 ± 17	65 ± 40	33 ± 10	32 ± 4
(E,Z)‐2,4‐Decadienal	25152‐83‐4	1308	1275 Van Durme et al. (2013)	Fried fatty geranium green waxy	19 ± 2	18 ± 10	14 ± 9	15 ± 9	26 ± 11	18 ± 8
Tetradecanal	124‐25‐4	1625	1906 Supelco Bulletin 923 (1998)	Fatty waxy amber incense dry citrus peel musk	8 ± 2	9 ± 8	10 ± 4	92 ± 62	9 ± 4	7 ± 2
Total					63	67	68	172	68	57
Alcanes										
Dodecane	112‐40‐3	nd	1200 Supelco Bulletin 923 (1998)	Alkane	55 ± 8	46 ± 10	43 ± 9	46 ± 15	71 ± 34	46 ± 6
5‐Ethyl 2,2,3 trimethylheptane	62199‐06‐8	1111	1110 Supelco Bulletin 923 (1998)	nd	26 ± 5	15 ± 7	16 ± 4	22 ± 5	36 ± 13	17 ± 9
Tetradecane	629‐59‐4	1150	1400 Supelco Bulletin 923 (1998)	Mild waxy	22 ± 9	27 ± 16	17 ± 8	16 ± 7	31 ± 9	35 ± 16
Total					103	88	76	84	138	98
Terpenes										
4‐(2,6,6‐Trimethyl‐1‐cyclohexenyl)‐3‐buten‐2‐one (β‐Ionone)	14901‐07‐6	1321	1493 Supelco Bulletin 923 (1998) 1488 Van Durme et al. (2013)	Floral woody sweet fruity berry tropical beeswax	41 ± 11	29 ± 18	40 ± 24	39 ± 41	40 ± 37	51 ± 33
1,3‐Di‐tert‐butylbenzene	1014‐60‐4	1224	1420 Supelco Bulletin 923 (1998)	nd	68 ± 33	88 ± 30	106 ± 15	148 ± 51	139 ± 59	85 ± 32
(2E,7R,11R)‐3,7,11,15‐Tetramethyl‐2‐hexadecenol (phytol)	150‐86‐7	1401	2000 Supelco Bulletin 923 (1998)	Delicate floral balsam powdery waxy	2188 ± 859	4073 ± 1019	1861 ± 1012	3137 ± 2839	2842 ± 192	3628 ± 149
Total					2297	4190	2007	3324	3021	3764
Alcohols										
3,7‐Dimethylocta‐1,6‐dien‐3‐ol (linalool)	78‐70‐6	1464	1526 Supelco Bulletin 923 (1998)	Citrus floral sweet bois de rose woody green blueberry	12 ± 10	10 ± 3	12 ± 3	16 ± 10	10 ± 5	13 ± 7
Tetradecanol (myristyl alcohol)	112‐72‐1	1795	nd Supelco Bulletin 923 (1998)	Fruity waxy orris coconut	6 ± 3	14 ± 11	16 ± 8	7 ± 3	38 ± 21	6 ± 1
Total					18	24	28	23	48	19
Acid										
Octadecanoic acid (stearic acid)	57‐11‐4	1403	3181 Supelco Bulletin 923 (1998)	Odorless mild fatty	1500 ± 211	864 ± 193	484 ± 352	3159 ± 2404	2102 ± 1259	468 ± 128

One of the most abundant volatile groups was the terpene group. (2E,7R,11R)‐3,7,11,15‐Tetramethyl‐2‐hexadecenol (phytol) was found in high amounts varying from 2188 μg/L for sample A to 4073 μg/L for sample B. This molecule is a decomposition product of chlorophyll (Belitz et al., 2004). In fact, chlorophyll is the most abundant pigment in *Chlorella vulgaris* (1–2 % dry weight) (Safi et al., 2014). Phytol is a lipophilic molecule, and its extraction is generally associated with lipids (Safi et al., 2014). This could explain the high levels in octadecanoic acid (stearic acid) extracted in samples: from 468 μg/L (sample F) to 3159 μg/L (sample D). Phytol has no odor or a very delicate one (The_good_scents_company, 2017). Phytol was already identified in microalgae (*Synechocystic sp*.) and in macroalgae (*Himanthalia elongata*). Antimicrobial activities of phytol have been proposed (Plaza et al., 2010).

Several alkanes were present in the microalgae samples. Dodecane seemed to be prevalent. According to Zhou et al. (2017), dodecane and tetradecane were in top 20 volatile components which significantly determine the differences between exponential phase and stationary phase of *Chlorella vulgaris*. Alkanes could derivate from lipid auto‐oxidation processes via alkyl radicals or from the decomposition of carotenoids. Similarly, aldehydes were quantified in the same order of magnitude. As described previously, the aldehydes could be the major products of the oxidation of fatty acids (Hidalgo & Zamora, 2007).

**Figure 2:**
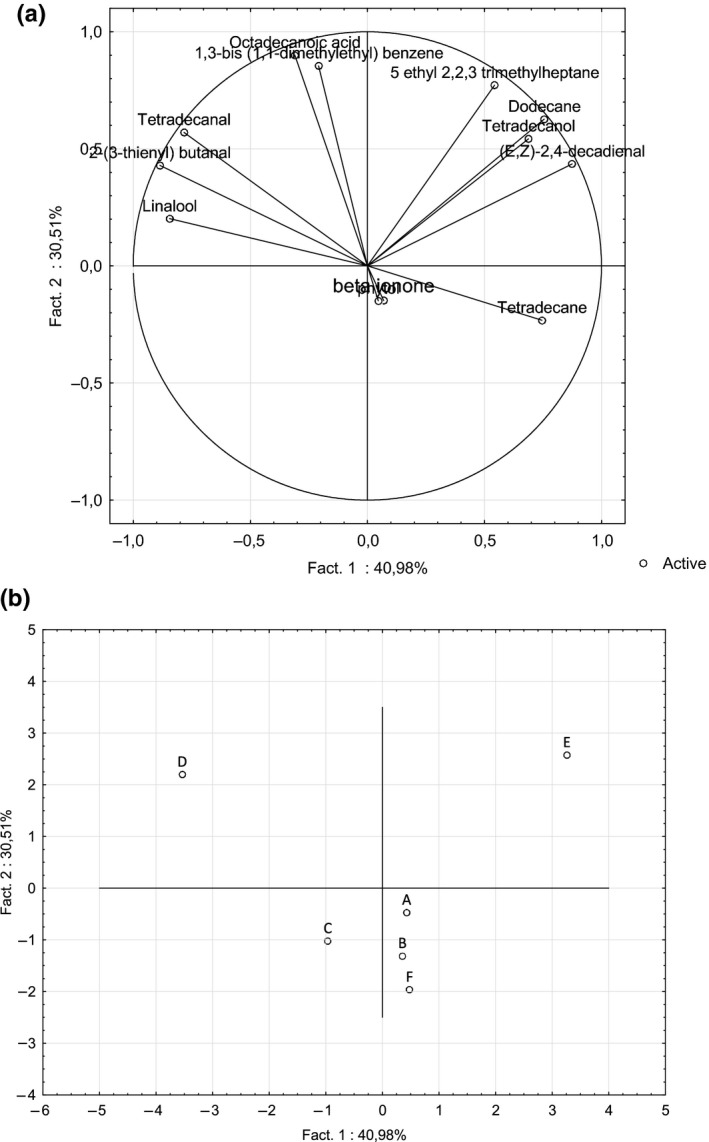
Principal component analysis (PCA) of volatile compounds identified in different samples of C*hlorella vulgaris* obtained by ultrasound‐assisted liquid–liquid extraction. (a) correlation circle of variables. (b) two‐dimensional projection of samples

Principal component analysis (PCA) was carried out to display the similarities and differences in volatile compounds detected among samples. The first two principal components were sufficient to explain 72 % of total variance (Figure 2a). The first and second principal components explained a variance of 41 % and 31 %, respectively. Excepted for β‐ionone and phytol, the vectors of variables were well close to the correlation circle in this two‐dimensional representation (Figure 2a). Based on PCA results, samples D and E were well differentiated from the other one depending on the volatile compounds identified (Figure 2b). Sample D was characterized by 2‐(3‐thienyl) butanal, tetradecanal (characteristic odor: fatty waxy, amber, incense, dry citrus peel, musk (The_good_scents_company, 2017)), and linalool (characteristic odor: citrus, floral, woody (The_good_scents_company, 2017)). On the other hand, sample E was characterized by dodecane (characteristic odor: alkane (The_good_scents_company, 2017)), 5‐ethyl 2,2,3 trimethylheptane, (E,Z)‐2,4‐decadienal (characteristic odor: geranium, green waxy (The_good_scents_company, 2017)), and tetradecanol (myristyl alcohol, characteristic odor: fruity, waxy, coconut (The_good_scents_company, 2017)). In this two‐dimensional representation, it is difficult to describe the volatile compounds profiles of the samples A, C, B, F.

With the ultrasound‐assisted LLE, only two samples were well different from the other counterparts: samples D and E. These two samples of *Chlorella vulgaris* showed each a different and typical volatile compounds profile. These results were different than those obtained by the SPME method and highlighted the effect of different extraction techniques on the volatile compounds profile obtained from same samples of *Chlorella vulgaris*.

## 4 DISCUSSION

Multivariate data analyses, performed separately for each extraction method, were useful to have a global picture of differences between the six samples of microalgae. The results highlighted that sample D had each a typical profile in volatile compounds, different from the other samples whatever the extraction method. Samples B, F, and E presented a typical profile only with one of the two extraction methods.

However, for a same given sample (D), the profiles of volatile compounds obtained were very different using the two extraction methods. Only one compound has been found in common for both extraction methods: β‐ionone. It seems so interesting to combine several extraction methods in order to obtain a complete view of volatile compounds profile.

**Figure 3:**
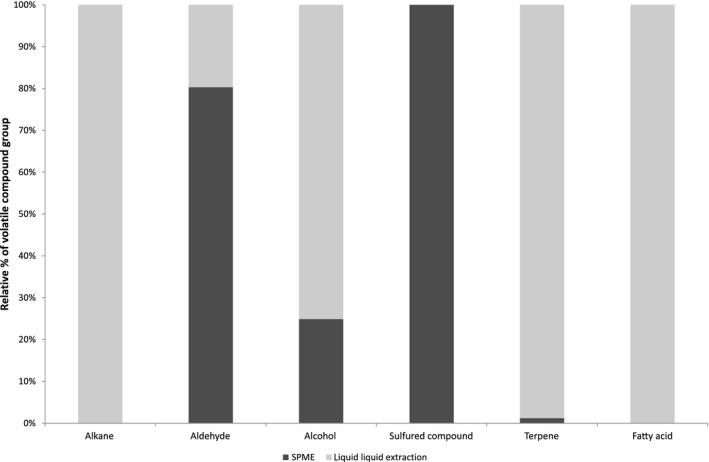
Comparison of chemical classes of the volatile compounds identified in different samples of C*hlorella vulgaris* using ultrasound‐assisted liquid–liquid extraction and SPME. Bars represent the relative percentage of the total amount of identified volatile compounds, grouped by chemical class

Figure 3 represents the chemical classes of volatile compounds identified for each extraction method. When using the SPME method, the main chemical classes of volatile compounds identified were as follows in decreasing order amount: sulfuric compounds, aldehydes (80%), alcohols (25%), and finally terpenes (1%). When using ultrasound‐assisted LLE, the profile was a much more balanced mixture of volatile compounds. The main chemical classes of volatile compounds identified were as follows in decreasing order amount: alkane, terpene, fatty acid, alcohol (75%), and aldehyde (20%).

**Figure 4:**
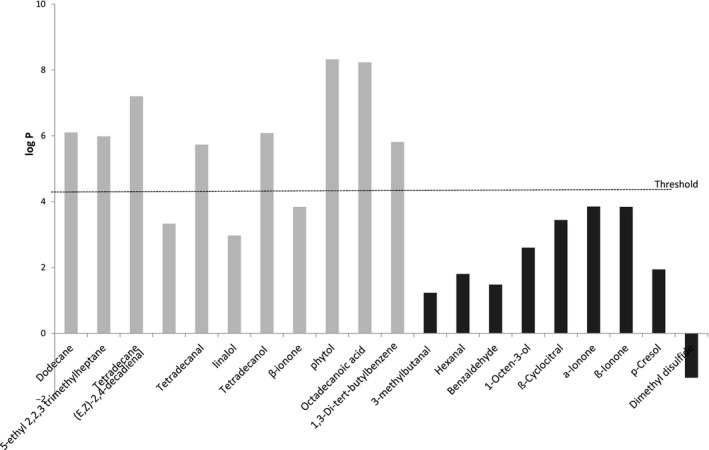
Relationship between log P of volatile compounds and the amount of identified volatile compounds according to the extraction method: SPME (in black) and ultrasound‐assisted liquid–liquid extraction (in gray)

In an attempt to explain this result, the relationships between the physicochemical properties (log P, boiling point) of volatile compounds identified and the mode of extraction used were studied. Figure 4 plots the log P distribution of the volatile compounds depending on the two modes of extraction. From these results, log P around 3 seemed to be a critical value. Below this value, the volatile compounds, whatever the chemical class, were better extracted with SPME than by LLE. On the opposite, the volatile compounds with a log P higher than 3 seemed to be more extracted by ultrasound‐assisted LLE than by SPME.

**Figure 5:**
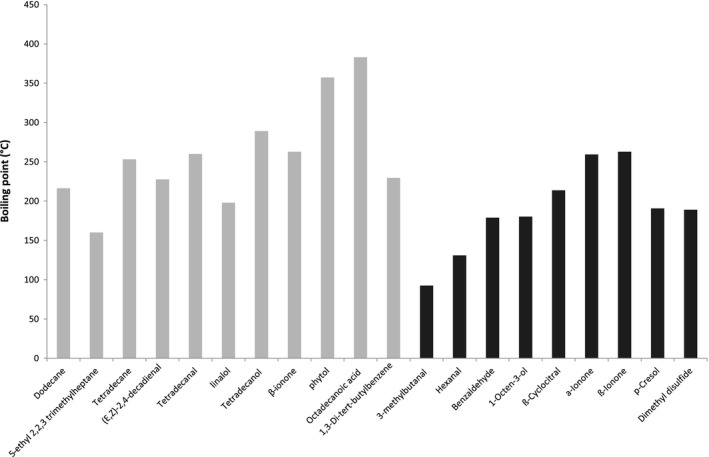
Relationship between boiling point of volatile compounds and the amount of identified volatile compounds according to the extraction method: SPME (in black) and ultrasound‐assisted liquid–liquid extraction (in gray)

The same reasoning was made with the boiling point (Figure 5). Conclusions were less clear, but a threshold boiling point value can be fixed around 160°C. Above this threshold boiling point value, the volatile compounds were extracted mainly by the ultrasound‐assisted LLE. Below this threshold boiling point value, the volatile compounds were extracted mainly by SPME. The vapor pressure of volatile compound and their solubility in water (data no shown) did not allow discriminating the two extraction methods for their extracting ability.

Consequently, the ultrasound‐assisted LLE method allowed the extraction of volatile compounds having a high boiling point (>160°C) and a high log P (>3) as compared to the SPME method. These results were consistent with a previous work done on a fat‐free model food system (Cayot et al., 2016). Indeed, the higher the boiling point and the log P values, the higher the extraction yield of volatile compounds with the LLE. The binary azeotrope mixture used in this study could then be a safe and good alternative solvent to extract volatile compounds whatever the composition of the matrix.

The SPME method was more effective to extract volatile compounds with a low boiling point (<160°C) and a low log P (<3). In the SPME method, the efficiency of the volatile compound adsorption onto the fiber can be affected by the fiber composition (Gamero, Wesselink, & de Jong, 2013). The general rule “similar is dissolved in similar” can be applied to SPME too, that is, polar compounds are sorbed on polar or semi‐polar fiber (CAR or DVB coating) and non‐polar compounds on non‐polar ones (PDMS coating) (Gamero et al., 2013; Wardencki et al., 2004). The SPME method can reach detection limits of 5–50 pg/g (Wardencki, Michulec, & Curyło, 2004). The fiber used in this study is the fiber recommended for the analysis of flavors and fragrances. (Supelco Bulletin 923, 1998).

Finally, the effectiveness of the SPME technique could be further increased by increasing the salt content in the sample resulting in a “salting‐out effect.”

As reported in the literature, results show that oxidation of lipids and proteins generates the majority of volatile compounds. It could so be recommended to limit the oxidation phenomena during the storage of microalgae and during the extraction of volatile compounds by avoiding exposure to light, oxygen, and high temperature.

## ETHICAL STATEMENTS

The authors declare that they do not have any conflict of interest. This study does not involve any human or animal testing.
